# 4558 Investigating the functional consequences of anaplastic lymphoma kinase (ALK) mutations arising upon Lorlatinib treatment

**DOI:** 10.1017/cts.2020.74

**Published:** 2020-07-29

**Authors:** Gabriela Maria Witek, Whelton Miller, David Slochower, Esther Berko, Yael Mossé, Mark Lemmon, Ravi Radhakrishnan

**Affiliations:** 1 University of Pennsylvania School of Medicine

## Abstract

OBJECTIVES/GOALS: Neuroblastoma (NB) is an embryonal cancer of the sympathetic nervous system that affects mostly infants and young children. The complex genetic background present across NB patients results in diverse clinical response and difficulty in individualizing therapy. Currently, NB patients undergo a regimen of genotoxic chemotherapeutics, radiation therapy, and new immunotherapy that, while effective, has significant side effects, including excruciating pain. One promising avenue for targeted therapy in neuroblastoma focuses on anaplastic lymphoma kinase (ALK), a cell surface neural receptor tyrosine kinase. We previously identified activating point mutations within the tyrosine kinase domain of ALK as the primary cause of hereditary NB, and we and others subsequently showed that these same alterations are the most common somatic single-nucleotide mutations in the sporadic forms of the disease. Crizotinib, a first-generation small molecule ATP-competitive inhibitor of the ALK tyrosine kinase, showed limited anti-tumor activity in patients with relapsed NB harboring ALK F1174 and F1245 mutations. We have demonstrated that lorlatinib, a novel ATP-competitive ALK inhibitor, overcomes this de novo resistance in preclinical models of ALK-driven NB. Recent clinical trials with lorlatinib in patients with non-small cell lung cancer harboring an ALK fusion, and in patients with NB harboring ALK mutations show the emergence of multiple or compound ALK mutations as a mechanism of resistance. We postulate that these compound mutations disrupt the interaction between and ALK and cause resistance. In this study, we employ a computational approach to model mutated ALK in complex with lorlatinib as well as ATP to understand whether the new mutations alter the affinity or mode of lorlatinib/ATP binding to ALK, and thus cause suboptimal ALK inhibition. METHODS/STUDY POPULATION: We employ methods in computational structural biology and drug design, primarily based on molecular modeling, molecular dynamics (MD), and molecular docking. Based on existing crystal structures of wildtype ALK, we model the mutations and perform MD simulations in order to characterize the activation state of the protein as well as perform ensemble docking calculations to assess the binding affinities and modes in ALK-lorlatinib and ALK-ATP complexes. RESULTS/ANTICIPATED RESULTS: We expect that the compound mutations cause resistance to lorlatinib either by lowering protein affinity for the drug or increasing the affinity for ATP. Alternatively, the compound mutations may disrupt the protein activation state, in which case ALK may no longer be active, and another protein/pathway could be driving the resistance. DISCUSSION/SIGNIFICANCE OF IMPACT: The results of this study will enable the understanding of the mechanism of resistance to lorlatinib and facilitate the design of new ALK inhibitors, or help develop more optimal and mechanism-guided therapies aimed to overcome the resistance.

